# A controlled weight loss intervention study among women of Somali background in Norway

**DOI:** 10.1016/j.jmh.2024.100231

**Published:** 2024-05-04

**Authors:** Linn Bohler, Haakon E. Meyer, Hein Stigum, Maria J. Leirbakk, Danielle Cabral, Mia Charlott Wedegren, Eivind Andersen, Mark L. Wieland, Ahmed A. Madar

**Affiliations:** a Department of Community Medicine and Global Health, Institute of Health and Society, University of Oslo, PO Box 1130 Blindern, 0316 Oslo, Norway; b Norwegian Institute of Public Health, 0213 Oslo, Norway; c Oslo Municipality, District Sagene, Vitaminveien 4, 0485 Oslo, Norway; d Oslo Municipality, District Gamle Oslo, Kolstadgata 1, 0652 Oslo, Norway; e Faculty of Humanities, Sports and Educational Science, Department of Sports, Physical Education and Outdoor Studies, University of South-Eastern Norway (USN), Post office box 4, 3199 Borre, Norway; f Center for Health Equity and Community Engagement Research, Mayo Clinic, Rochester, MN 55902, USA; g Division of Community Internal Medicine, Geriatrics, and Palliative Care, Mayo Clinic, Rochester, Minnesota 55902, USA

**Keywords:** Obesity, Lifestyle, Body weight, Immigrants

## Abstract

**Background:**

Women of Somali background in Norway have a high prevalence of overweight and obesity, compared with women in the general Norwegian population. For lifestyle interventions to be applicable for immigrants to Norway, it is important to culturally tailor interventions in collaboration with relevant communities. The primary outcome was a difference in weight change between the intervention and control groups.

**Methods:**

In this interventional study, Somali women living in one borough of Oslo Municipality in Norway with body mass index (BMI) ≥27.0 kg^.^*m*^-^^2^ received a co-created, culturally tailored 12-month weight loss intervention consisting of 24 interactive sessions during the first three months, and monthly sessions for the next nine months, compared to a control group. Both groups were measured at baseline and 12 months.

**Results:**

A total of 169 participants were recruited, and 101 participants completed the follow-up. After multiple imputation, the mean difference in weight change adjusted for baseline weight, age, education, employment, marital status, number of children in the household and length of Norwegian residency was -1.6 kg (95 % CI -3.57, 0.43, *p* = 0.12) in the intervention compared to the control group.

**Conclusion:**

This culturally tailored intervention study demonstrated a modest non-significant effect on weight change after 12 months, possibly due to the short intervention duration and COVID-19. Further studies of a longer duration and considering the provision of childcare are needed to understand whether this approach can be transferred to other immigrant groups and genders.

Trial registration: The study was registered at clinicaltrials.gov NCT04578067, 2020-09-29.

## Highlights

• An intervention study developed in collaboration with Somali user representatives.
• Women-only, female instructors, and the use of the Norwegian and Somali languages.
• Emphasis on lifestyle change through self-empowerment and self-efficacy.
• Modest non-significant effect on weight change after 12 months.
• Further studies should be of longer duration.

## Graphical abstract

**Figure:**
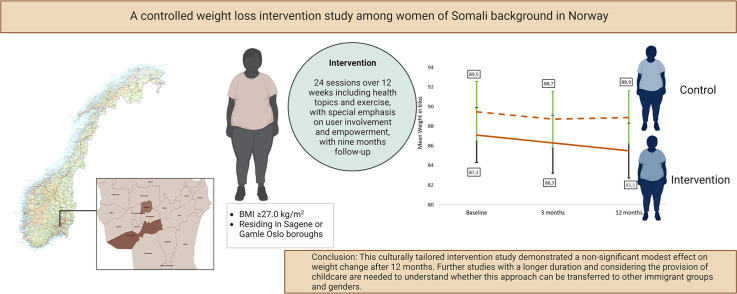

## 1 Introduction

In Norway and the Oslo Municipality, immigrants accounted for 16 % and 26 % of the population in January 2023, respectively (SSB 2023). People of Somali background represent one of the largest non-European immigrant groups in Norway (SSB 2023).

The prevalence of overweight and obesity among female immigrants is high compared to ethnic Norwegians, but there are large differences between immigrant groups (Kumar et al., 2006). A previously conducted comparative study showed that approximately 80 % of women of Somali background in Norway were overweight or obese (Ahmed et al., 2018), approximately 70 % reported physical inactivity and/or a sedentary lifestyle and one-third reported poor health status. Being overweight and obese increases the risk of non-communicable diseases such as type 2 diabetes, cardiovascular disease, and some cancers (WHO 2021; WHO 2023). Additionally, there is an association between increasing musculoskeletal disability and obesity (Wearing et al., 2006).

There is a lack of research on prevention programs and treatment for obesity and chronic diseases among immigrants to high income countries (Renzaho et al., 2010). Moreover, the representation of minority populations in research is scarce, especially among African immigrant populations (Turk et al., 2018). Most of the previous research on obesity prevention in immigrant populations has focused mainly on Latinos and has been constrained by methodological limitations (Renzaho et al., 2010; Turk et al., 2018; Tovar et al., 2014). However, culturally focused interventions that utilised engagement strategies and community resources, such as bilingual workers, demonstrated positive effects for addressing obesity in both adults and children (Tovar et al., 2014). Additionally, previous studies suggest that for lifestyle interventions to be applicable in immigrant groups of different cultures, they should focus on behavioural change through increased self-empowerment and self-efficacy (Lirussi, 2010). Moreover, the intervention should be gender-specific and should consider differences in preferences for physical activity (Lirussi, 2010; Jaber et al., 2011).

Programs culturally tailored for people of immigrant backgrounds have been developed in Australia and the US (Tovar et al., 2012; Renzaho et al., 2015). In recent years, some studies have been conducted that address immigrants to the developed world (Renzaho et al., 2010; Wieland et al., 2018; Telle-Hjellset et al., 2013; Siddiqui et al., 2017). However, there remains an urgent public health imperative to develop interventions with and for immigrant groups in different contexts to address the complex structural, environmental, social, and behavioural barriers to healthful weight loss and weight maintenance in this group (Siddiqui et al., 2017).

Prior to the present study, a mixed-methods study was conducted as a collaboration between academic partners, the Oslo Municipality, and Somali community partners, which found that many women of Somali background changed their diet when they moved to Norway (Madar et al., 2023). Moreover, barriers to healthful eating habits included high food costs, language difficulties and children's influence on the family's eating habits (Madar et al., 2023). Their understanding of the benefits of physical activity was good, but mixed-gender gyms, the cost of a gym membership, family responsibilities, and the weather were considered barriers to exercise (Madar et al., 2023). To help address the barriers identified in this study, the women offered suggestions for a culturally tailored lifestyle intervention program for women of Somali background, which resulted in the design of the current study (Madar et al., 2023).

This study aimed to assess the effect of a culturally tailored intervention package (physical activity, behavioural health, and nutrition education) among overweight and obese women of Somali background. The study examined changes in body weight and cardio-metabolic outcomes from baseline to 12 months in an intervention group compared to a control group receiving usual care.

## 2 Material and methods

### 2.1 Study setting

The study took place at the Healthy Life Centres in two boroughs in the municipality of Oslo. These boroughs were selected because they had among the highest proportions (5.9 % and 3.5 %) of immigrants of Somali background, and well-functioning Somali women networks, representing and serving the Somali population.

### 2.2 Study design

This was an interventional study design compared with a control group and based on the data obtained from the Increased Physical Activity and a Healthier Lifestyle among Immigrant Women study (ASLI) (clinicaltrials.gov NCT04578067). The study aimed to explore the impact of a culturally adapted lifestyle programme on weight reduction.

### 2.3 Population, sample, and groups compared

Between September 2020 and May 2023, participants were enrolled from two boroughs in Oslo, Norway, through several channels, including Somali radio, women's groups, general practitioners, and female user representatives from Somali community-based organisations. The representatives were included in the study team to facilitate information and recruitment. Eligible participants were overweight or obese (body mass index (BMI) ≥27 kg^.^*m*^-^^2^) adult women of Somali background, aged 20 years and above, who lived in Oslo, Norway.

The exclusion criteria were pregnancy at the time of enrolment, active participation in a formal weight loss program, serious musculoskeletal problems or difficulty walking, significant medical co-morbidities, like cancer or uncontrolled diabetes, taking medications that may affect weight loss or suffering from a diagnosed eating disorder. Those who fulfilled the eligibility criteria were included in the study. The women were recruited through Somali radio, women's groups, general practitioners, and female user representatives from Somali community-based organisations, who were included in the study team helping with information and recruitment.

Randomisation at the individual level was not feasible due to the risk of contamination. Allocation of treatment conditions was therefore assigned between the two boroughs’ Healthy Life Centres described above. In order to decide which borough would receive the intervention and which would be the control, a draw was made. Inhabitants in the intervention borough were allocated to the intervention group and inhabitants in the control borough were allocated to the control group.

A control group of Somali women from a different borough received a delayed intervention after 12 months. No restrictions or expectations were given concerning behaviour change, and, if received, standardised care from the borough's health services (lifestyle advice, and information on diet as well as exercise) could persist. During the 12-month control period, the participants were contacted regularly by SMS from the study team, to sustain contact.

### 2.4 Intervention

The 12-month intervention programme was developed following the aforementioned informative study (Madar et al., 2023) and in collaboration with Somali user representatives from the two boroughs. It was designed as a combination of group education and coaching on physical activity, sedentary behaviour, healthy diet, and weight loss strategies.

The core component of the programme was to counsel and empower women through individual counselling and group education sessions with an emphasis on physical activity and the creation of an empowering environment. The goal was to examine the effect of a lifestyle programme, including physical activity and health topics twice per week, on weight change in the intervention compared to a control group. The intervention group received 24 sessions, distributed twice a week for the first 12 weeks with interactive sessions of 90 min and then monthly for nine months held by the study group. To fit with the women's schedule, and possibly increasing adherence, two timepoints per session day were provided, one at midday and one in the evening. The 45-minute sessions included interactive content on healthy eating (cooking, meal sizes, compositions, beverages, reading of food labels and keyhole products, smart shopping), physical activity, sedentary behaviour, and weight loss strategies using SMART goals (specific, measurable, achievable, relevant, time-based). The group sessions included 45 min of low-threshold physical activity with low to moderate intensity (resistance exercise, cycling, yoga, balance, or walking) at the Healthy Life Centres training rooms or outdoors in surrounding areas with instructors.

### 2.5 Measures

The primary outcome was the difference in weight change in kilograms between the intervention and control groups after 12 months. Weight was measured to the nearest 0.1 kg by an electric Omron medical scale (BF214, Hoofdorp, The Netherlands). Secondary outcomes were changes in blood pressure as measured by an automatic blood pressure measuring device (Omron HBP-1320; Hoofddorp, The Netherlands), changes in the blood concentration of glycated haemoglobin (HbA1c, mmol/mol and %), and changes in the blood concentration of cholesterol (total cholesterol (TC), low-density lipoprotein (LDL), and high-density lipoprotein (HDL) mmol/L) measured by non-fasting blood tests between intervention and control group after 12 months. Waist circumference was measured by Seca 201 (Hamburg, Germany) measuring tape at the midpoint between the lower margin of the last palpable rib and the top of the iliac crest to the nearest 0.1 cm, with the participant standing and breathing normally. Attendance was measured at every session by the coach crossing off the participant's name on the attendance list. After the intervention, the participants filled out a questionnaire involving satisfaction with the programme and reading nutritional declarations when grocery shopping. In addition, the questionnaire also contained questions on perceived health after the intervention.

### 2.6 Data collection

The study took place between September 2020 and May 2023 and was designed to include three health examinations, at baseline, after three and 12 months. All measurements were conducted by a trained study team who followed and used standardised protocols and tools. Each assessment included a physical examination, collection of blood samples, and questionnaires, as previously described in detail (Bohler et al., 2023). Before starting the intervention, a risk analysis was carried out due to the coronavirus pandemic to identify potential risks and how they should be managed if they occurred. The study was carried out during the coronavirus pandemic, and social distancing and coronavirus restrictions affected attendance at data collection and the intervention.

The same project team, which consisted of a nutritionist, nurse, epidemiologist, and public health expert performed the baseline and follow-up data collection at both boroughs. A bilingual and culturally congruent researcher managed the project, and the communication language was Somali, but most of the study participants were able to communicate in the Norwegian language as well. Data analysts were blinded to the treatment condition throughout the study and when analysing the data.

### 2.7 Statistical methods

Differences at baseline between the intervention and control groups were compared using an independent sample *t*-test for normally distributed or Mann-Whitney *U* test for non-normally distributed continuous variables, chi-square test, or Fisher's exact test for categorical variables (Table 1).

**Table 1:** Baseline characteristics of the participants in the intervention and the control group.

Variables	Intervention (*n* = 82)	Control (n = 87)	p-value
Age (years), mean (SD)	45.8 (10.1)	48.0 (10.9)	0.201^a^
Education ≤10 years, n (%)	59 (72.0)	59 (68.6)	0.635^b^^,^^g^
Unemployment, n (%)^d^	35 (42.7)	36 (41.4)	0.437^b^^,^^e^
Married, n (%)	43 (52.4)	62 (72.1)	0.041^b^^,^^f^
Number of children in the household, mean (SD)	2.2 (1.8)	2.3 (1.9)	0.875^c^^,^^h^
Body mass index (BMI, kg.*m*^-^^2^), mean (SD)	33.6 (5.0)	34.2 (5.2)	0.444^a^
Waist circumference (cm), mean (SD)	99.2 (10.5)	97.3 (10.8)	0.259^a^
Systolic blood pressure (mmHg), mean (SD)	123.1 (17.7)	122.9 (19.9)	0.611^c^
Diastolic blood pressure (mmHg), mean (SD)	81.3 (10.3)	81.8 (9.8)	0.739^a^
Non-fasting glucose (mmol/L), mean (SD)	6.3 (1.4)	6.4 (1.7)	0.781^c^
HbA1c (mmol/mol), mean (SD)	38.7 (6.7)	39.1 (8.6)	0.694^c^
HbA1c (%), mean (SD)	5.7 (0.6)	5.7 (0.8)	0.381^c^
Total cholesterol (mmol/L), mean (SD)	4.9 (0.9)	5.0 (0.9)	0.619^a^
LDL (mmol/L), mean (SD)	2.4 (0.7)	2.5 (0.9)	0.221^i^
HDL (mmol/L), mean (SD)	1.4 (0.3)	1.6 (0.7)	0.055^c^^,^^j^

Differences between groups were compared using independent samples *t*-test for continuous variables and chi-square test for categorical variables and Mann Whitney *U test* for variables that were not normally distributed.

a Independent samples *t*-test.

b Chi-square test.

c Mann-Whitney *U test*.

d Unemployment is defined as currently unemployed, retired, disabled, leave of absence and sick leave.

e Examined the difference in overall employment in the two groups.

f Examined the difference in overall marital status in the two groups.

g Intervention group *n* = 82, control group *n* = 86.

h Intervention group *n* = 82, control group *n* = 86.

i Intervention group *n* = 81, control group *n* = 84.

j Intervention group *n* = 82, control group *n* = 86.

A statistician blinded for the grouping of the participants, and who was not otherwise involved in the study, executed the main analyses. Multiple linear regression was used to assess weight change (primary outcome) from baseline to 12 months with adjustment for baseline weight (Holmberg and Andersen, 2022) and additional adjustment for age, education, employment, marital status, number of children in the household and length of Norwegian residence. Analysis of secondary outcomes (BMI, waist, blood lipids, HbA1c (mmol/mol and %), non-fasting glucose, and blood pressure) was performed in the same manner.

A multiple imputation with predictive mean matching was executed, assuming data is missing at random, and aiming to reduce bias and improve precision (White et al., 2022). With this approach, the distribution of the observed data is used to estimate multiple possible values for the data points (White et al., 2022). For multiple imputation and predictive mean matching, 40 imputations were used, reflecting the percentage of participants with a missing outcome, and ten was used as the number of nearest neighbours for predictive mean matching. A multiple imputation plot with an explanation is attached as supplement material, Fig. A1. Statistical analysis was performed using STATA (version 17, StataCorp. LLC, Texas, USA).

**Fig. 1:**
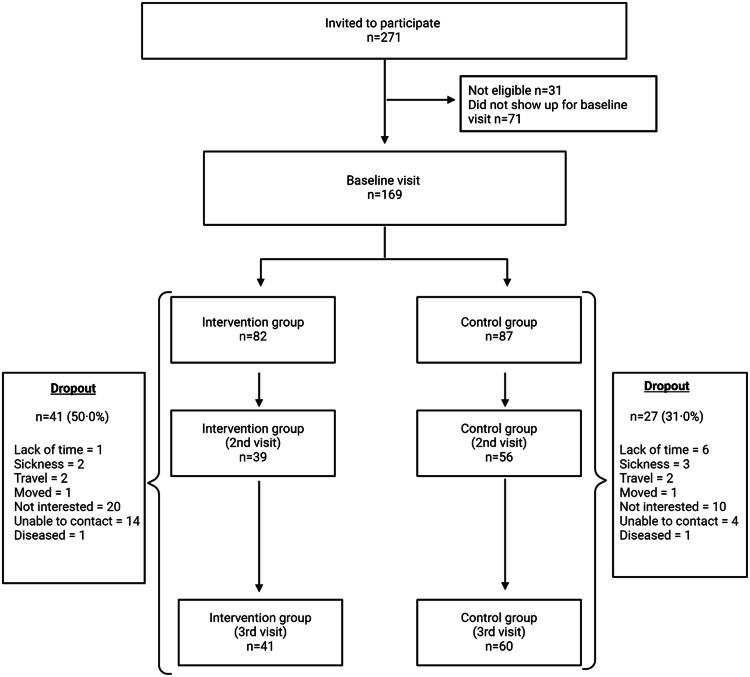
Flow chart of the study participants.

An a priori sample size estimation determined that 64 participants were required in each group to provide 80 % power to detect a between-group mean (SD) difference of 2.5 (5.0) kilos at 12 months, with a significance level of 0.05. Due to an expected dropout rate of 25 %, the study aimed to include 200 participants.

### 2.8 Ethical considerations

This study was conducted according to the guidelines established in the Declaration of Helsinki and was approved by the Norwegian Centre for Research Data (NSD) (ref: 724,880). The study was registered in clinicaltrials.gov NCT04578067.

All participants provided written consent before participating in the study. There were no elements of the data collection that posed a known health risk to the women. The participants were informed about their results and referred for further follow-up as appropriate.

## 3 Results

Of 271 women of Somali background invited to participate, 169 accepted to participate, attended the first visit, and were found eligible. Fig. 1 shows a flow chart of the study participants.

There were significantly more married women in the control group compared to the intervention group (Table 1). Variations were observed for other variables, but these differences were not statistically significant (Table 1). Similar results were discovered for complete case participants (*n* = 101) shown in the supplement material (Table A1).

A total of 68 participants (40.2 %) in the intervention and control groups were lost to follow-up between baseline and 12 months: 50 % in the intervention group and 31 % in the control group. The dropouts differed in education length, and HDL, but did not differ in weight, employment status, age, marital status, number of children in the household, systolic blood pressure, diastolic blood pressure, HbA1c (mmol/mol or %), non-fasting glucose, total cholesterol, or LDL, compared to those who completed the study. In response to an open-ended question, the primary reasons for dropping out were lack of interest, lack of time, and illness.

### 3.1 Primary outcome

The mean weight in the intervention group was 87.1 kgs at baseline and 85.5 kg at follow-up (mean change of −1.6 kg), while the mean weight in the control group was 89.5 kg at baseline and 88.9 kg at follow-up (mean change of −0.6 kg), after multiple imputation. A complete case analysis can be found in the supplement materials (Table A2).

**Table 2:** Multiple linear regression results for primary and secondary endpoints after multiple imputations (*n* = 169).

	Mean treatment difference (95 %CI)^a^	Adjusted mean treatment difference (95 %CI)^b^
**Primary endpoint**		
Δweight	−1.44 (−3.33, 0.45)	−1.57 (−3.57, 0.43)
**Secondary endpoints**		
ΔBMI	−0.53 (−1.25, 0.20)	−0.58 (−1.35, 0.18)
ΔWaist	−2.16 (−4.99, 0.67)	−1.91 (−4.82, 1.00)
ΔHba1c (mmol/mol)	−0.60 (−2.25, 1.06)	−0.56 (−2.27, 1.14)
ΔHba1c (%)	−0.05 (−0.20, 0.11)	−0.04 (−0.20, 0.12)
ΔNon-fasting glucose	−0.32 (−0.98, 0.35)	−0.25 (−0.95, 0.44)
ΔSystolic BP	−1.42 (−7.68, 4.85)	−1.03 (−7.44, 5.37)
ΔDiastolic BP	1.02 (−2.62, 4.67)	0.46 (−3.32, 4.25)
ΔTotal cholesterol	0.06 (−0.20, 0.32)	0.08 (−0.20, 0.36)
ΔLDL	0.17 (−0.10, 0.44)	0.16 (−0.12, 0.44)
ΔHDL	0.02 (−0.10, 0.14)	0.01 (−0.11, 0.14)

a Adjusted for baseline value.

b Adjusted for age, education, employment, marital status, number of children in the household, and length of Norwegian residency.

The intervention program consisted of 24 sessions for 12 weeks. After multiple imputations, there was a mean difference in weight change of −1.4 kg in the intervention group compared to the control group after adjusting for baseline weight (Table 2). Additionally, after additional adjustment for age, education, employment, marital status, length of Norwegian residency, and number of children in the household the mean difference in weight change was −1.6 kg in the intervention group compared to the control group (Table 2). In the complete case analysis, a mean difference of −1.5 kg (95 % confidence interval −2.96, 0.06, *p* = 0.06) and a mean difference of −1.6 kg (95 % confidence interval −3.21, 0.08, *p* = 0.06) after adjusting, were discovered between the intervention and control groups (Supplement materials, Table A2).

**Fig. 2:**
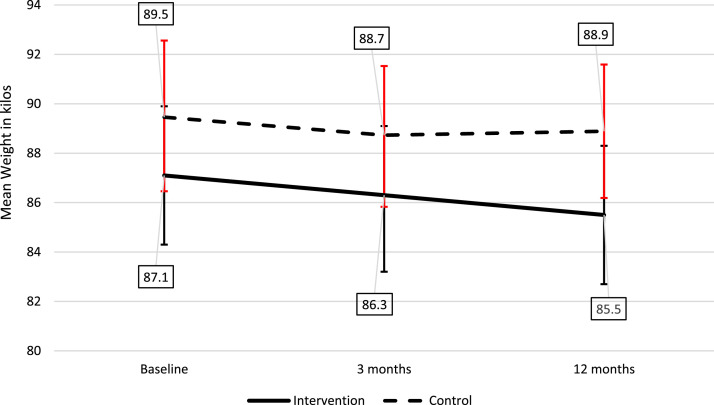
Weight change from baseline to 12 months in the intervention (*n* = 82) and control groups (*n* = 87) after multiple imputation shown as mean with confidence intervals.

In the intervention group, 14.6 % sustained 3 % weight loss at 12 months compared with 12.6 % in the control group (*p* = 0.16), adjusted for baseline weight, age, marital status, education, employment, number of children in the household, and length of Norwegian residence). Fig. 2 shows the weight change over time between the intervention and control groups after multiple imputations (*n* = 169). The participants in the intervention group (*n* = 82) had a steady weight loss from baseline to 12 months. In the control group (*n* = 87), mean weight decreased from baseline to three months and slightly increased from three to 12 months (Fig. 2).

### 3.2 Secondary outcomes

The mean difference in BMI adjusted for baseline BMI was −0.5 (95 % confidence interval −1.25, 0.20) between the intervention and control groups. When adjusting for age, education, employment, marital status, length of Norwegian residence, and the number of children in the household a difference of −0.6 (95 % confidence interval −1.35, 0.18) was discovered. There was no difference in change for waist circumference, HbA1c (mmol/mol or %), non-fasting glucose, systolic and diastolic blood pressure, or lipids from baseline to 12 months between the groups (Table 2). A complete case analysis can be seen in the supplement material (Table A2).

The median attendance in the intervention programme was 9.5 times. Additional analysis on weight change and intervention attendance with linear regression showed no significant effect.

In the questionnaire filled out postintervention, 65.9 % reported always reading the food and nutrition declaration when grocery shopping. The questionnaire also included questions on perceived health after the intervention, with 80.5 % reporting feeling in better shape than before the intervention. After the intervention, 95 % of the women who participated in the programme would recommend the program to others. Additionally, they gave the programme an average rating of 9.7 (0.5) out of 10, and similarly rated the coaches at 9.7 (0.5).

## 4 Discussion

In this culturally tailored intervention programme developed with and for women of Somali background in Norway, the primary analysis showed a modest non-significant weight loss in the intervention group compared to the control group. Moreover, no significant difference in waist circumference, blood pressure, HbA1c, non-fasting glucose or lipids between the intervention and control groups were detected. In addition, the women reported increased health literacy and that they felt they were in better shape after participating in the study.

To our knowledge, this is among the first culturally tailored intervention studies on Somali or Sub-Saharan immigrants in Europe. The difference in weight change between the intervention and control groups in our study is modest and was not statistically significant. Only two previous culturally tailored intervention studies have addressed people of Somali background (Wieland et al., 2018; Wikström et al., 2021). They revealed no significant differences in weight (Wikström et al., 2021) or BMI (Wieland et al., 2018; Wikström et al., 2021) at follow-up. However, a Finnish study on people of Somali background had relatively few participants and short duration (Wikström et al., 2021). In addition, in a study among Hispanic, Somali, and Sudanese immigrant families in Minnesota, weight loss was not a primary outcome (Wieland et al., 2018).

Moreover, it has been previously reported that participating in a lifestyle intervention study, even in a control group, motivates healthier behaviour and weight change (Johns et al., 2016). This may have prevented a significant difference in weight change between the intervention and control groups in the US study and the present study. Additionally, the coronavirus pandemic had a great impact on the completion of the study and led to a lot of absenteeism and dropouts. However, it is possible that participating occasionally led to positive social network effects among women in the intervention borough, and thereby contributed to weight loss over time in this group (Njeru et al., 2020; Fletcher et al., 2011). This is supported by a continued weight loss from three to 12 months in our study.

Weight loss has been evaluated as an outcome of some culturally adapted lifestyle interventions globally, but mainly in Hispanic populations (Rosas et al., 2020; Ockene et al., 2012; Parikh et al., 2010; Van Name et al., 2016; Wadden et al., 2009). Some studies on immigrant populations have found that the intervention group lost more weight than the control group at 12 months (Siddiqui et al., 2017; Rosas et al., 2020; Ockene et al., 2012; Parikh et al., 2010; Van Name et al., 2016; Wadden et al., 2009; Katzmarzyk et al., 2020; Ash et al., 2006), and no change in waist circumference (Wieland et al., 2018; Siddiqui et al., 2017; Rosas et al., 2020; Ash et al., 2006). In some of these studies, a difference in weight change of less than three kilos was reported (Siddiqui et al., 2017; Rosas et al., 2020; Ockene et al., 2012; Ash et al., 2006) and some studies reported a difference in weight loss of more than three kilos (Parikh et al., 2010; Van Name et al., 2016; Wadden et al., 2009; Katzmarzyk et al., 2020). Moreover, the studies were performed on participants of another background than Somali, with higher body weight at baseline, and some aimed at treating or preventing diabetes in participants of high risk (Siddiqui et al., 2017; Rosas et al., 2020; Ockene et al., 2012; Parikh et al., 2010; Van Name et al., 2016; Wadden et al., 2009; Katzmarzyk et al., 2020; Ash et al., 2006), and the studies are therefore not directly comparable to the present study.

The participants were generally very satisfied with the execution and content of the programme and would recommend it to others. However, we encountered some challenges during the implementation, which have given us valuable learning to consider in future prevention activities for women of Somali background and other minority groups. Even though we offered sessions in the middle of the day and the evening twice a week (i.e., four times per week), the participants had difficulties with attendance. The low adherence to the programme was probably mainly due to the coronavirus pandemic and, the responsibility for children living at home and school or work with varying time schedules. In the study on people of Somali background in Finland, only 50 % participated in the group sessions, despite having agreed on the timetable and being conducted in the mosque (Wikström et al., 2021). This was due to a lack of time or conflicting timetables (Wikström et al., 2021). As a solution, they suggest further studies to provide childcare during the group sessions (Wikström et al., 2021). On the contrary, the study from the US had a good adherence but still failed to show a significant difference in weight after twelve months (Wieland et al., 2018). However, there was a relatively large but non-significant effect at six months, but this effect was not sustained at 12 months (Wieland et al., 2018).

In addition to the relatively poor attendance rate and that following the whole programme may have produced different results, this study also had a large dropout. The dropout in the US and Finnish studies were low compared to the present study. However, the lack of significant results could be caused by the short duration of these studies and the present study. A study from the UK discovered that it takes an average of 1.5 times longer to establish a physical activity habit than it does to for example to do small changes in diet (Lally et al., 2010; Arlinghaus and Johnston, 2019). Additionally, habit formation varied greatly among individuals, ranging from 18 to 254 days (Lally et al., 2010). Therefore, it is crucial to customise health advice and treatment expectations individually (Arlinghaus and Johnston, 2019; Noar et al., 2007; Khoury et al., 2016) and to implement interventions of longer duration.

Methodological aspects of the research design could also have prevented a significant difference in weight between the two groups. It varied how much clothes they were willing to remove at each weighing, but the participants reported to wear the same amount at each measurement. If there is a discrepancy between the amount of clothes worn, it could have prevented the study from reaching a significant difference in weight between the two groups. Additionally, as the participants belong to the same community across Oslo, we cannot rule out intervention contamination in the control group. The risk of contamination across groups was also reported in the US study (Wieland et al., 2018).

No improvement in non-fasting glucose, HbA1c, TC, HDL, LDL, waist, or blood pressure were discovered in this study. Similar results have been reported in other studies (Wieland et al., 2018; Telle-Hjellset et al., 2013; Siddiqui et al., 2017; Wikström et al., 2021; Parikh et al., 2010; Van Name et al., 2016; Rosas et al., 2015). At baseline, the mean values were mostly within normal ranges, consequently with a lower potential for improvement at follow-up.

### 4.1 Strengths and limitations

The participants in our study, like other studies, were not excluded based on native language proficiency (Jaber et al., 2011; Wieland et al., 2018; Telle-Hjellset et al., 2013; Siddiqui et al., 2017; Wikström et al., 2021; Rosas et al., 2020; Ockene et al., 2012; Parikh et al., 2010; Van Name et al., 2016; Wadden et al., 2009; Katzmarzyk et al., 2020), due to a Somali-speaking researcher in the study team and Somali user representatives in both boroughs and could therefore be more representative of the Somali population. In a study from the US, to reach people of Latino background, only Spanish-speaking participants were included (Rosas et al., 2015). Contrarily, a study from Australia excluded potential participants based on English proficiency (Ash et al., 2006). Data collection was performed by the same study team at baseline and follow-up. Other strengths were the participatory development of a culturally adapted group-based approach including nutrition, learning a quick and easy way of baking whole meal bread, barriers and facilitators to lifestyle change, and health topics. In addition, a female trainer at the Healthy Life Centre was a central element and a strength of this study. Participants in the study are assumed to represent overweight and obese women of Somali background in Oslo, but selection bias cannot be completely ruled out as those who participated may be more motivated and connected to the community, and potentially healthier than those who did not participate (Juul, 2012).

This study had limitations. First, the major limitation of this study is that it was conducted during the coronavirus pandemic, and adherence to the intervention was difficult due to quarantine, being infected with COVID-19, and occasional lockdowns of schools and kindergartens. Second, this study had a high dropout rate which made the study underpowered, and we cannot rule out a larger (or smaller) effect size if retention was higher. Multiple imputations were used to address missing data but can only be used if the data is missing at random, however, we cannot rule out that the data is not missing at random. The effect after multiple imputations was quite similar to the complete case, but since the imputed sets varied to some extent, the uncertainty was somewhat greater, meaning that predicting missing values was challenging. Third, the intervention season is a limitation, as it mainly took place during the autumn and winter months in order to avoid the Ramadan period (the fasting month of Muslims), which is known to influence dietary and physical activity habits (Siddiqui et al., 2017). Fourth, the participants were asked to wear as little as possible at weight measurements, but it varied how much clothes they were willing to remove. However, participants did agree to wear the same amount of clothing at each weighing. Fifth, the study was not randomised at the individual level, which is not possible in activity studies as the participants will know if they exercise or not, but since the participants knew which groups they were allocated to, it could have affected the expectations and the outcome. Sixth, even though the participants lived in different boroughs, we cannot rule out the possibility of contamination between the intervention- and control boroughs due to being part of the same community across the city of Oslo and increased use of social media during the COVID-19 period (Hunter et al., 2015).

## 5 Conclusion

This culturally tailored intervention study for women of Somali background had a modest non-significant effect on weight change after 12 months, possibly due to the short intervention duration and COVID-19. Given that women of Somali background are highly represented among adults in Norway with obesity, it is critical to pursue future research aimed at increasing the effectiveness of behavioural lifestyle interventions for the prevention of non-communicable diseases. Further studies with a longer duration and considering the provision of childcare are needed to understand whether this approach can be transferred to other immigrant groups and genders.

## CRediT authorship contribution statement

**Linn Bohler:** Writing – review & editing, Writing – original draft, Visualization, Validation, Software, Resources, Project administration, Methodology, Investigation, Formal analysis, Data curation. **Haakon E. Meyer:** Writing – review & editing, Validation, Supervision, Project administration, Methodology, Formal analysis, Conceptualization. **Hein Stigum:** Writing – review & editing, Validation, Supervision, Formal analysis, Data curation. **Maria J. Leirbakk:** Writing – review & editing, Project administration, Investigation, Data curation. **Danielle Cabral:** Writing – review & editing, Investigation, Data curation. **Mia Charlott Wedegren:** Writing – review & editing, Resources, Project administration, Methodology, Funding acquisition, Conceptualization. **Eivind Andersen:** Writing – review & editing, Project administration, Methodology, Conceptualization. **Mark L. Wieland:** Writing – review & editing, Project administration, Methodology, Conceptualization. **Ahmed A. Madar:** Writing – review & editing, Validation, Supervision, Resources, Project administration, Methodology, Investigation, Funding acquisition, Formal analysis, Conceptualization.

## Declaration of competing interest

The authors declare that they have no known competing financial interests or personal relationships that could have appeared to influence the work reported in this paper.

## Appendix Supplementary materials

Image, application 1

## References

[bib0001] Ahmed S.H., Meyer H.E., Kjollesdal M.K., Madar A.A. (2018). Prevalence and Predictors of Overweight and Obesity among Somalis in Norway and Somaliland: a Comparative Study. J. Obes.

[bib0002] Arlinghaus K.R., Johnston C.A. (2019). The Importance of Creating Habits and Routine. Am. J. Lifestyle. Med.

[bib0003] Ash S., Reeves M., Bauer J., Dover T., Vivanti A., Leong C. (2006). A randomised control trial comparing lifestyle groups, individual counselling and written information in the management of weight and health outcomes over 12 months. Int. J. Obes. (Lond).

[bib0004] Bohler L., Meyer H.E., Leirbakk M.J., Wedegren M.C., Rangsvag H.G., Kjollesdal M.K. (2023). Risk factors for non-communicable diseases among overweight and obese women of Somali background in Oslo. Norway Clin. Epidemiol. Global Heal.

[bib0005] Fletcher A., Bonell C., Sorhaindo A. (2011). You are what your friends eat: systematic review of social network analyses of young people's eating behaviours and bodyweight. J. Epidemiol. Commun. Heal.

[bib0006] Holmberg M.J., Andersen L.W. (2022). Adjustment for baseline characteristics in randomized clinical trials. JAMA.

[bib0007] Hunter R.F., McAneney H., Davis M., Tully M.A., Valente T.W., Kee F. (2015). Hidden" social networks in behavior change interventions. Am. J. Public Heal.

[bib0008] Jaber L.A., Pinelli N.R., Brown M.B., Funnell M.M., Anderson R., Hammad A. (2011). Feasibility of group lifestyle intervention for diabetes prevention in Arab Americans. Diabetes. Res.. Clin.. Pract..

[bib0009] Johns D.J., Hartmann-Boyce J., Jebb S.A., Aveyard P. (2016). Weight change among people randomized to minimal intervention control groups in weight loss trials. Obesity.

[bib0010] Juul S., Ostergaard B, Skogemann L (2012). Epidemiologi Og Evidens.

[bib0011] Katzmarzyk P.T., Martin C.K., Newton R.L., Apolzan J.W., Arnold C.L., Davis T.C. (2020). Weight loss in underserved patients - a cluster-randomized trial. N. Engl. J. Med.

[bib0012] Khoury M.J., Iademarco M.F., Riley W.T. (2016). Precision public health for the era of precision medicine. Am. J. Prev. Med.

[bib0013] Kumar B.N., Meyer H.E., Wandel M., Dalen I., Holmboe-Ottesen G. (2006). Ethnic differences in obesity among immigrants from developing countries, in Oslo, Norway. Int. J. Obes.

[bib0014] Lally P., van Jaarsveld C.H.M., Potts H.W.W., Wardle J. (2010). How are habits formed: modelling habit formation in the real world. Eur. J. Soc. Psychol.

[bib0015] Lirussi F. (2010). The global challenge of type 2 diabetes and the strategies for response in ethnic minority groups. Diabetes Metab. Res. Rev.

[bib0016] Madar A.A., Marie Brux C., Wedegren M.C., Rangsvåg H., Ek N.L., Thompson A.L. Barriers and facilitators to physical activity and healthy eating: a qualitative study among Somali women in Oslo, Norway. Norsk tidsskrift for ernæring. 2023;21(1):7–17. 10.18261/ntfe.21.1.3.

[bib0017] Njeru J.W., Wieland M.L., Okamoto J.M., Novotny P.J., Breen-Lyles M.K., Osman A. (2020). Social networks and obesity among Somali immigrants and refugees. BMC Public. Heal.

[bib0018] Noar S.M., Benac C.N., Harris M.S. (2007). Does tailoring matter? Meta-analytic review of tailored print health behavior change interventions. Psychol. Bull.

[bib0019] Ockene I.S., Tellez T.L., Rosal M.C., Reed G.W., Mordes J., Merriam P.A. (2012). Outcomes of a latino community-based intervention for the prevention of diabetes: the lawrence latino diabetes prevention project. Am. J. Public Health.

[bib0020] Parikh P., Simon E.P., Fei K., Looker H., Goytia C., Horowitz C.R. (2010). Results of a pilot diabetes prevention intervention in East Harlem, New York City: project HEED. Amer. J. Public Health..

[bib0021] Renzaho A.M., Halliday J.A., Mellor D., Green J. (2015). The Healthy Migrant Families Initiative: development of a culturally competent obesity prevention intervention for African migrants. BMC Public Health.

[bib0022] Renzaho A.M., Mellor D., Boulton K., Swinburn B. (2010). Effectiveness of prevention programmes for obesity and chronic diseases among immigrants to developed countries - a systematic review. Public Health Nutr.

[bib0023] Rosas L.G., Lv N., Xiao L., Lewis M.A., Venditti E.M.J., Zavella P. (2020). Effect of a culturally adapted behavioral intervention for latino adults on weight loss over 2 years: a randomized clinical trial. JAMA. Netw. Open.

[bib0024] Rosas L.G., Thiyagarajan S., Goldstein B.A., Drieling R.L., Romero P.P., Ma J. (2015). The Effectiveness of Two Community-Based Weight Loss Strategies among Obese, Low-Income US Latinos. J. Acad. Nutr. Diet.

[bib0025] Siddiqui F., Kurbasic A., Lindblad U., Nilsson P.M., Bennet L. (2017). Effects of a culturally adapted lifestyle intervention on cardio-metabolic outcomes: a randomized controlled trial in Iraqi immigrants to Sweden at high risk for Type 2 diabetes. Metabolism.

[bib0026] SSB. Immigrants and Norwegian-born to immigrant parents Oslo: statistisk sentralbyrå; 2023 [updated March 6 2023,. Available from: https://www.ssb.no/en/befolkning/innvandrere/statistikk/innvandrere-og-norskfodte-med-innvandrerforeldre.

[bib0027] Telle-Hjellset V., Raberg Kjollesdal M.K., Bjorge B., Holmboe-Ottesen G., Wandel M., Birkeland K.I. (2013). The InnvaDiab-DE-PLAN study: a randomised controlled trial with a culturally adapted education programme improved the risk profile for type 2 diabetes in Pakistani immigrant women. Br. J. Nutr.

[bib0028] Tovar A., Renzaho A.M.N., Guerrero A.D., Mena N., Ayala G.X. (2014). A systematic review of obesity prevention intervention studies among immigrant populations in the US. Curr. Obes. Rep.

[bib0029] Tovar A., Vikre E.K., Gute D.M., Kamins C.L., Pirie A., Boulos R., et al. Development of the live well curriculum for recent immigrants: a community-based participatory approach. progress in community health partnerships: research, education, and action. 2012;6(2):195–204. 10.1353/cpr.2012.0024. PMC3511891 22820229

[bib0030] Turk M.T., Kalarchian M.A., Nolfi D.A., Fapohunda A. (2018). Prevention and treatment of overweight and obesity among African immigrant populations: a systematic review of the literature. Annu. Rev. Nurs. Res.

[bib0031] Van Name M.A., Camp A.W., Magenheimer E.A., Li F., Dziura J.D., Montosa A. (2016). Effective translation of an intensive lifestyle intervention for hispanic women with prediabetes in a community. Health Center Setting Diabet. Care..

[bib0032] Wadden T.A., West D.S., Neiberg R.H., Wing R.R., Ryan D.H., Johnson K.C. (2009). One-year weight losses in the look AHEAD study: factors associated with success. Obesity.

[bib0033] Wearing S.C., Hennig E.M., Byrne N.M., Steele J.R., Hills A.P. (2006). Musculoskeletal disorders associated with obesity: a biomechanical perspective. Obes. Rev.

[bib0034] White I.R., Pandis N., Pham T.M. (2022). Missing data, part 5. Introduction to multiple imputation. Am. J. Orthodon. Dentofac. Orthop.

[bib0035] WHO. Obesity and overweight 2021 [Available from: https://www.who.int/news-room/fact-sheets/detail/obesity-and-overweight.

[bib0036] WHO (2023). https://www.who.int/news-room/fact-sheets/detail/noncommunicable-diseases.

[bib0037] Wieland M.L., Hanza M.M.M., Weis J.A., Meiers S.J., Patten C.A., Clark M.M. (2018). Healthy immigrant families: randomized controlled trial of a family-based nutrition and physical activity intervention. Am. J. Health. Promot.

[bib0038] Wikström K., Hussein I., Virtanen E., Nekouei Marvi Langari M., Mattila E., Lindström J (2021). Culturally sensitive lifestyle intervention to prevent type 2 diabetes among Somalis in Finland: a pilot study using JA CHRODIS recommendations and criteria. Ann. Ist. Super. Sanita.

