# Supplementary material for: A controlled weight loss intervention study among women of Somali background in Norway
Source: J Migr Health. 2024 May 4;9:100231. doi: 10.1016/j.jmh.2024.100231 (PMC11101690; doi:10.1016/j.jmh.2024.100231)
Supplement: Supplementary file 1 [file mmc1.docx]

**Supplementary materials**

**Contents**

[Baseline characteristics 2](#_Toc151558798)

[Table A1 Baseline characteristics of the participants in the intervention and the control group, complete case 2](#_Toc151558799)

[Multiple imputations 3](#_Toc151558800)

[Figure A1 Forest plot with a complete case of 101 observations (blue line), five randomly selected imputed sets and the multiple imputation effect with 169 observations (green line) 3](#_Toc151558801)

[Primary outcome, complete case analysis 4](#_Toc151558802)

[Table A2 Changes from baseline to 12 months for primary and secondary outcomes for the intervention group (n=41) and control group (n=60), complete case 4](#_Toc151558803)

# **Baseline characteristics**

There were significantly more married women in the control group compared to the intervention group in the complete case (Table A1). Variations were observed for other variables, but these differences were not statistically significant (Table A1).

### **Table A1 Baseline characteristics of the participants in the intervention and the control group, complete case**

| **Variables** | **Intervention (n=41)** | **Control (60)** | **p-value** |
| --- | --- | --- | --- |
| Age (years), mean (SD) | 45·6 (9·3) | 47·9 (9·0) | 0·233 |
| Education ≤10 years, n (%) | 27 (65·9) | 37 (61·7) | 0·519_ab_ |
| Unemployment, n (%)_c_ | 17 (41·5) | 25 (41·7) | 0·147_d_ |
| Married, n (%) | 20 (48·9) | 46 (76·7) | 0·011_e_ |
| Number of children in household, mean (SD) | 2·2 (1·9) | 2·2 (2·0) | 0·953_a_ |
| Body mass index (BMI, kg^.^m^−2^), mean (SD) | 33·1 (5·2) | 33·9 (5·1) | 0·471 |
| Waist circumference (cm), mean (SD) | 97·2 (9·2) | 97·2 (10·8) | 0·969 |
| Systolic blood pressure (mmHg), mean (SD) | 125·0 (19·3) | 123·9 (19·5) | 0·782_a_ |
| Diastolic blood pressure (mmHg), mean (SD) | 82·2 (11·0) | 81·7 (9·4) | 0·789 |
| Non-fasting glucose (mmol/L), mean (SD) | 6·3 (1·4) | 6·5 (1·8) | 0·655_a_ |
| HbA1c (mmol/mol), mean (SD) | 38·3 (5·9) | 39.0 (9·1) | 0·776_a_ |
| HbA1c (%), mean (SD) | 5·7 (0·5) | 5·7 (0·8) | 0·605_a_ |
| Total cholesterol (mmol/L), mean (SD) | 4·9 (0·9) | 5·0 (1·0) | 0·899 |
| LDL (mmol/L), mean (SD) | 2·4 (0·7) | 2·5 (0·9) | 0·590_f_ |
| HDL (mmol/L), mean (SD) | 1·5 (0·3) | 1·7 (0·8) | 0·082_ag_ |

Differences between groups were compared using independent samples t-test for continuous variables and chi-square test for categorical variables.

_a_ Mann-Whitney U-test
_b_ Examined the difference between overall education lengths in the two groups
_c_ Unemployment is defined as currently unemployed, retired, disabled, leave of absence and sick leave
_d_ Examined the difference in overall employment in the two groups
_e_ Examined the difference in overall marital status in the two groups
_f_ Intervention group n=41, control group n=57
_g_ Intervention group n=41, control group n=59

# **Multiple imputations**

The plot (Figure A1) shows the effect of the intervention on weight change as a coefficient with the confidence interval from an adjusted regression. Line 1 shows the complete case with 101 observations. Here the effect is borderline significant (*p*=0·06). The next five lines show the effects in five randomly selected imputed sets from the 40 imputed sets of 169 observations. They show the variation between the imputed sets.

The last line shows the multiple imputation effect with 169 observations and 40 imputed sets. The effect here is quite similar to the complete case, but since the imputed sets vary quite a bit, the uncertainty here is somewhat greater.


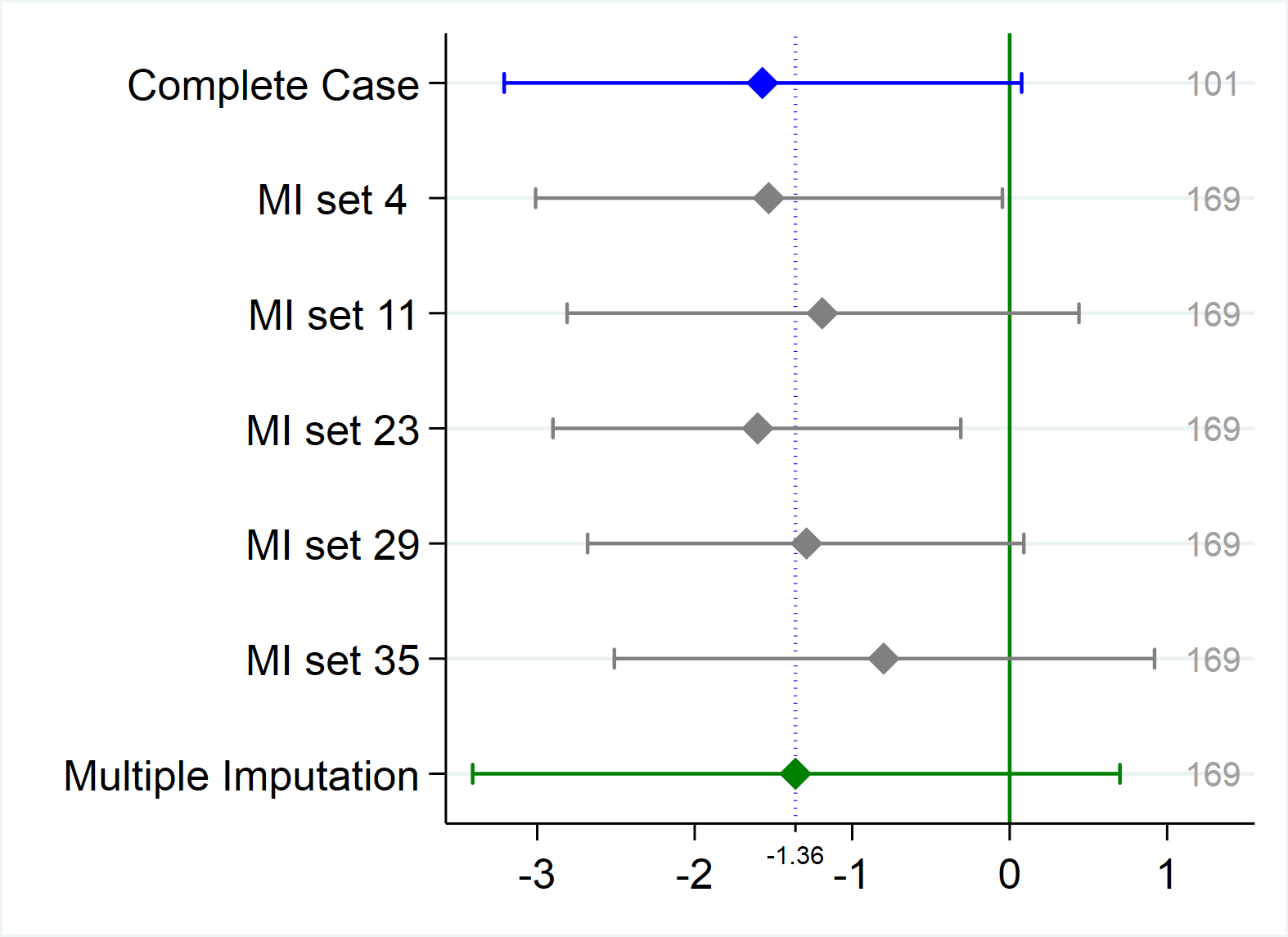


### **Figure A1 Forest plot with a complete case of 101 observations (blue line), five randomly selected imputed sets and the multiple imputation effect with 169 observations (green line)**

# **Primary outcome, complete case analysis**

The mean weight in the intervention group was 87·1 kilos (kg) at baseline and 84·8 kg at follow-up (mean change of -0·9 kg), while the mean weight in the control group was 89·5 kg at baseline and 88·5 kg at follow-up (mean change of +0·3 kg) (Table A2).

The mean difference in weight change adjusted for baseline weight was -1·5 kg (95% confidence interval -2·96–0·06, *p*=0·06) in the intervention group versus the control group for complete case analysis. After additional adjustment for age, education, employment, marital status, length of Norwegian residency and number of children in the household the mean difference in weight change was -1·6 kg (95% confidence interval -3·21–0·08, *p*=0·06) in the intervention group versus the control group.

### **Table A2 Changes from baseline to 12 months for primary and secondary outcomes for the intervention group (n=41) and control group (n=60), complete case**

|  | **Baseline, mean (SD)** | **12 months, mean (SD)** | | **Δ change from baseline to 12 months, mean (SD)** | **Mean treatment difference (95%CI)_a_** | **Adjusted mean treatment difference(95%CI)_b_** |
| --- | --- | --- | --- | --- | --- | --- |
| **Primary** |  |  | |  |  |  |
| Weight (kg) |  |  | |  |  |  |
| Control | 89·5 (14·5) | 88·5 (12·5) | | 0·29 (3·5) |  |  |
| Intervention | 87·1 (12·9) | 84·8 (13·0) | | -0·88 (4·7) |  |  |
| Δweight |  |  | |  | -1·45 (-2·96–0·06) | -1·57 (-3·21–0·08) |
| **Secondary** |  |  | |  |  |  |
| BMI (kg^.^m^−2^) |  |  | |  |  |  |
| Control | 34·2 (5·2) | 33·9 (4·7) | | 0·1 (1·3) |  |  |
| Intervention | 33·6 (5·0) | 32·8 (4·8) | | -0·4 (1·8) |  |  |
| ΔBMI |  |  | |  | -0·56 (-1·13–0·02) | -0·59 (-1·21–0·03) |
| Waist (cm) |  |  | |  |  |  |
| Control | 97·3 (10·8) | 98·4 (9·2) | | 1·2 (6·5) |  |  |
| Intervention | 99·2 (10·5) | 96·4 (10·5) | | -0·8 (5·9) |  |  |
| Δwaist |  |  | |  | -2·05 (-4·39–0·30) | -1·96 (-4·34–0·42) |
| HbA1c (mmol/mol) |  |  | |  |  |  |
| Control | 39·1 (8·6) | 40·6 (11·4) | | 1·6 (3·1) |  |  |
| Intervention | 38·7 (6·7) | 39·8 (9·5) | | 1·5 (4·6) |  |  |
| ΔHba1c (mmol/mol) |  |  | |  | 0·08 (-1·14–1·31) | -0·01 (-1·35–1·32) |
| HbA1c (%) |  |  | |  |  |  |
| Control | 5·7 (0·8) | 5·9 (1·0) | | 0·1 (0·3) |  |  |
| Intervention | 5·7 (0·6) | 5·8 (0·9) | | 0·1 (0·4) |  |  |
| ΔHba1c (%) |  |  | |  | 0·01 (-0·10–0·13) | 0·00 (-0·12–0·12) |
| Non-fasting Glucose (mmol/L) |  |  | |  |  |  |
| Control | 6·4 (1·7) | 6·7 (2·5) | | 0·1 (1·9) |  |  |
| Intervention | 6·3 (1·4) | 6·1 (1·2) | | -0·3 (1·8) |  |  |
| Δnon-fasting glucose |  | |  |  | -0·44 (-1·17–0·28) | -0·16(-0·93–0·61) |
| Systolic BP (mmHg) |  | |  |  |  |  |
| Control | 122·9 (19·9) | | 122·5 (19·2) | -1·5 (16·3) |  |  |
| Intervention | 123·1 (17·7) | | 121·9 (23·8) | -3·0 (20·6) |  |  |
| ΔSystolic BP |  | |  |  | -1·22 (-8·05–5·61) | -1·54 (-8·78–5·71) |
| Diastolic BP (mmHg) |  | |  |  |  |  |
| Control | 81·8 (9·8) | | 76·8 (9·5) | -4·9 (9·9) |  |  |
| Intervention | 81·3 (10·3) | | 79·0 (12·1) | -3·2 (11·7) |  |  |
| ΔDiastolic BP |  | |  |  | 1·92 (-1·87–5·71) | 0·32 (-3·70–4·34) |
| Total Cholesterol (mmol/L) |  | |  |  |  |  |
| Control | 5·0 (0·9) | | 4·9 (0·8) | -0·1 (0·7) |  |  |
| Intervention | 4·9 (0·9) | | 4·9 (0·7) | 0·0 (0·7) |  |  |
| Δtotal cholesterol |  | |  |  | 0·04 (-0·17–0·26) | 0·03 (-0·20–0·27) |
| LDL (mmol/L) |  | |  |  |  |  |
| Control | 2·5 (0·9) | | 2·2 (0·7) | -0·3 (1·0) |  |  |
| Intervention | 2·4 (0·7) | | 2·4 (0·8) | 0·0 (0·7) |  |  |
| ΔLDL |  | |  |  | 0·24 (-0·04–0·52) | 0·24 (-0·07–0·55) |
| HDL (mmol/L) |  | |  |  |  |  |
| Control | 1·6 (0·7) | | 1·6 (0·3) | -0·1 (0·8) |  |  |
| Intervention | 1·4 (0·3) | | 1·5 (0·3) | 0·1 (0·3) |  |  |
| ΔHDL |  | |  |  | -0·02 (-0·14–0·09) | -0·04 (-0·16–0·09) |

_a_Adjusted for baseline value
_b_Adjusted for age, education, employment, marital status, number of children in the household, and length of Norwegian residence
